# Medical and Surgical Society of Baltimore

**Published:** 1877-01

**Authors:** 


### MEDICAL AND SURGICAL SOCIETY OF BALTIMORE.

### FOREIGN BODY IN THE LUNG.

Dr. D. W. Cathell: About three months ago a youth, æt. 16 years, accidentally swallowed the head of a timothy straw about three inches long. The body became lodged behind the epiglottis; and the patient applied to a dentist, who pushed it down into the larynx, from whence it made its way into the right lung. About one week later he came under the care of a physician, who treated him for pneumonia; but as the physician shortly afterwards left the city, the treatment was not followed up. The patient now became hectic. The sputa became putrid and offensive, and occasional hemorrhage occurred. I was, at about this period, called to the case. On auscultation and percussion, found a vomica in the lower portion of the right lung, with considerable surrounding dullness. Prof. N. R. Smith was called in consultation, and gave the opinion that the foreign body was still in the lung. He, moreover, stated that he has seen some twenty cases where foreign bodies have made their way out of the lungs, and thought it not improbable that this case might eventually terminate in that way. It sometimes takes place even after the lapse of some years. In order to facilitate the escape of the body through the walls of the chest, the patient was placed on the right side in order to allow gravitation towards the surface; the chest was blistered over the vomica, and the patient was put on tonics and supporting treatment. My unbounded confidence in Prof. Smith's judgment and skill leads me to believe that this patient has an excellent chance of getting well.

Dr. Uhler: I have met with a case presenting a similar history to the one related by Dr. Cathell. After the supervention of hectic, etc., the patient was taken with a violent fit of coughing, and spat up a part of a chincapin. Heat, moisture, etc., ought to disintegrate the timothy, and thus facilitate its expulsion in the expectorated matter.

Dr. Cathell: I have immersed a head of timothy in the sputa expectorated by this boy, in order to determine how long it will take for its disintegration, but so far have not obtained any definite results.

Dr. Seldner: The German authors record a number of cases where foreign bodies have made their way out of the lungs and stomach by various means.

### ASCITES DUE TO THE ATROPHY OF THE LIVER AND ENLARGEMENT OF THE SPLEEN.

Dr. Arnold: A patient was brought to the hospital attached to the Washington University, to be treated for ascites. The abdomen was enormously enlarged. The patient had been exposed to malarial influences for a number of years, and the dropsy was attributed to the influence of malaria on the liver, kidneys, and spleen. Whilst it was not possible to determine by physical exploration the precise size of those organs, yet it was a manifest fact that the liver was atrophied and the spleen was enlarged. Paracentesis was performed, and after a quantity of serum was drawn off, the flow stopped, but percussion still revealed the existence of fluid. Ten days later the patient was again tapped, with a similar result. A few days later the patient died. The post-mortem examination showed the spleen to weigh forty-four ounces—while it will be remembered that its normal weight is about seven ounces. It was attached to the stomach and diaphragm, and its parenchyma was black and friable. There existed general peritonitis; the peritoneal cavity was subdivided by fibrinous bands into different compartments, thus constituting sacculated or encysted dropsy. An abscess was found between the spleen and pancreas. The liver was atrophied, the atrophy being confined more especially to the left lobe. A section of the liver did not reveal the nodulated aspect met with in hobnailed liver. It would be of some pathological interest to know whether the ascites was due to compression of the terminal branches of the vena portæ, and obstruction of the passage of blood through the liver, or to the peritonitis. I am inclined to the former opinion. In this case the fluid presented a clear appearance, while in ascites from peritoneal inflammation it exhibits a turbid appearance, with numerous flakes or flocculi of albumen.

### OVARIOTOMY.

Dr. Monmonier: I was recently called to visit a lady, and found a cystic tumor of the right ovary. The case was of long duration, and had attained considerable size. The subject was greatly emaciated, and every feature of the case promised an unfavorable prognosis; but at the earnest request of the patient and her friends I determined to operate. The patient was placed under the influence of chloroform, an incision was made through the abdominal walls and peritoneum, when two cysts attached to the right ovary were exposed. The contents of the cysts were evacuated and their sacs removed. In the absence of the clamp the pedicle was secured by means of the ordinary cat-gut ligature. Drainage was established through Douglas' cul-de-sac. Bands of lymph were found filling the abdominal cavity and agglutinating the intestines. After the operation perfect rest was enjoined, and an opiate was administered. The patient did well after the operation, and, contrary to expectations, made a rapid recovery.

Dr. Erich: Will Dr. Monmonier inform the Society what became of the ligature? I do not understand that there is any necessity for the use of the cat-gut ligature under the old supposition that it will become absorbed. The silver suture can be left in the wound, as it becomes encysted, and is not liable to produce sloughing of the stump. Would like to know how the ligature was removed: was it cut off or pulled away? Pulling on the ligature would have the effect of tearing the vessels in the pedicle and causing hemorrhage.

Dr. Monmonier: The ligature was left hanging through the vaginal wall, and a week after, when it had softened down, it was gradually pulled away. It was formerly supposed that the animal ligature was absorbed; it is now supposed to be changed in structure.

### TRAUMATIC NEURITIS.

Dr. A. B. Arnold presented the following case to illustrate the clinical history of neuritis: Last winter a girl was brought into the Washington University Hospital with a needle in her arm near the elbow. The needle was removed, and she was under treatment for six weeks afterwards. She suffered intense pain about the seat of injury; the joint became swollen, and there was also some contraction of the muscles of the arm, and anæsthesia and partial paralysis of the hand and fingers. An eruption of blisters or vesicles appeared on the arm. The median nerve could be traced like a cord up the arm. Concluded that the case was one of neuritis. The girl was highly hysterical, and had several more needles to be taken out. She frequently left the hospital, and while out would introduce the needles herself. She eventually became perfectly well.

### URÆMIA.

Dr. Caldwell: Infant, born healthy, but up to the second day had not voided its urine. On the second day it was seized with uræmic convulsions, and the case terminated fatally on the next day. In these cases what is the suppression of urine to be attributed to—the urethra, bladder, or kidneys?

Dr. Seldner: It is sometimes due to occlusion of the urethra or ureters, or to some congenital defect in the bladder or kidneys.

Dr. McShane: A case recently came under my care, in which the trouble was due to congenital phymosis, there being no opening through the fore-skin. Circumcision relieved the little patient.

Dr. Erich: I have met with three similar cases, all males, in which I succeeded in emptying the bladder by the introduction of a No. 1 gum catheter.

LACERATION OF THE POSTERIOR TIBIAL ARTERY.

Dr. Monmonier: A boy was attacked and bitten in the leg by a vicious dog. The posterior tibial artery was lacerated, profuse hemorrhage ensued, and to control the same the artery was ligated. Five days after the boy went out horseback-riding, and not returning at the expected hour, search was instituted, when he was found dead on the roadside. Death took place from hemorrhage. On cutting down on the artery, ulceration of the artery and detachment of the clot was found to have occurred. The laceration of the artery was two inches long.—*Physician and Surgeon.*

